# Development and validation of a predictive model for depression risk in Chinese obese adults

**DOI:** 10.3389/fpubh.2025.1574386

**Published:** 2025-05-14

**Authors:** Cong Yu, Jiamin Cao, Wenguang Chen, Ensi Hong

**Affiliations:** 1 Clinical Medical College, Jiangxi University of Chinese Medicine, Nanchang, China; 2 Nanchang Normal University, Infirmary of the Logistics Support Department, Nanchang, Jiangxi, China; 3 The Affiliated Hospital of Jiangxi University of Chinese Medicine, Nanchang, China

**Keywords:** obesity, risk of depression, predictive models, nomogram, Chinese population

## Abstract

**Objective:**

To construct a prediction model for the risk of depression in the obese population, aiming to facilitate the early identification of high-risk individuals and guide personalized preventive interventions.

**Methods:**

This study was based on the data from the China Health and Retirement Longitudinal Study (CHARLS 2015), the Center for Epidemiologic Studies Depression Scale-10 (CES-D10) to assess the depression of obese patients, Lasso regression and multivariable logistic regression were used to select predictors, the construction of a nomogram model, and the use of the random splitting method divided into a training set (*n* = 974) and a validation set (*n* = 418) by the 7:3 method, and the model was evaluated by the ROC curves and the AUC, the H-L goodness-of-fit test, the calibration graphs, and the clinical decision-making curve to assess the model.

**Results:**

A total of 1,392 obese patients were finally included, with a prevalence of depression of 32.68%. Age, respiratory function, renal disease, digestive disease, grip strength, rheumatism and arthritis, and sleep duration were selected to construct the predictive nomogram model of depression risk in obese patients, and the AUCs of the training set and validation set were 0.715 (95% CI = 0.681–0.749) and 0.716 (95% CI = 0.665–0.767). This suggests that the model has moderate discriminatory power. Respectively, the H-L test was statistically insignificant (*p* > 0.05, H-L test; *p* > 0.05). Goodness of fit, calibration curves showed significant agreement between the model and actual observations, and clinical decision curves indicated good model calibration and net benefit.

**Conclusion:**

The model constructed in this study has good efficacy in predicting the occurrence of depression in the obese population and can be used for the early identification of high-risk groups and the adoption of targeted preventive measures to reduce the risk of depression.

## 1 Introduction

Currently, obesity has become a major public health problem in China and worldwide (1). With the rapid social and economic development, the lifestyle and dietary structure of the population have undergone significant changes, and the prevalence of overweight and obesity among Chinese residents has shown a significant upward trend (2). In China, more than 50% of adults have overweight or obesity problems (3). Studies have shown that obesity increases the risk of hypertension, diabetes mellitus, coronary heart disease, stroke, specific cancers, osteoporosis, and many other chronic diseases (4–7), which seriously affects daily activities and quality of life. At the same time, obesity is closely associated with mood disorders, increasing the risk of depression and anxiety (8), and studies have shown that obese adults are 23–36% more likely to develop depression compared to non-obese people, and that an elevated body mass index (BMI) even predicts the chronic course of depression and anxiety symptoms (9). At the same time, obesity and depression are potentially linked in a multidimensional way. Current research has focused on inflammation, gut flora, GBA/microbiota-GBA, neuroplasticity and HPA axis abnormalities. Studies have shown that dysregulation of hormones such as leptin and adiponectin secreted by adipose tissue may affect brain neurotransmitters, leading to mood disorders (10, 11). Obesity-associated chronic low-grade inflammation can cross the blood–brain barrier and trigger neuroinflammation (12), directly impairing mood regulation. At the same time, both are associated with reduced intestinal flora diversity, and flora metabolites may affect mood and appetite regulation through the ‘gut-brain axis’ (13). Both obese and depressed patients have hyperactivation of the HPA axis, which leads to elevated cortisol levels (14, 15). Chronic high cortisol levels can damage the hippocampus and exacerbate depressive symptoms (16). In addition, in terms of shared genetic risk, some genes may increase susceptibility to both obesity and depression, with NEGR1 identified as the most important functional gene and associated with both at a genome-wide significance level (17).

The development of depression is reversible to some extent, including at the physical, psychological, and social support levels (18). Early screening and intervention in high-risk groups are essential to delay the onset and progression of depression. Risk prediction models are widely used in various diseases to identify the risk of developing depression in high-risk groups (19, 20). Previous studies have focused on investigating the status of depression and the factors that influence it, and little attention has been paid to developing risk prediction models to screen patients at high risk of depression in the obese population. This study aimed to identify factors associated with depression in obese patients and incorporate them into a nomogram constructed based on a model for predicting depression in obese patients. The constructed nomogram can help obese people to self-check whether they have high-risk factors for depression and intervene on their own. At the same time, based on the predictive model of the nomogram, clinical staff can quickly screen out patients with a high risk of depression in the obese population, thus providing a basis for the development of mental health education and prevention strategies for the obese population.

## 2 Methods

### 2.1 Study design

We used data from the China Health and Retirement Longitudinal Study (CHARLS), which is publicly available at http://charls.pku.edu.cn. It was approved by the Biomedical Ethics Committee of Peking University (Beijing, China). The data were of high quality and large sample nature, which provided real and effective data support for the analyses in this paper. Data from the CHARLS 2015 were selected for analysis in this study. Which included participants with a BMI ≥ 28 kg/m^2^, no key variables missing from the data, and age ≥18 years, after excluding participants with >20% missing data. 1,392 patients were included in the analysis. Our study was conducted by the Declaration of Helsinki. The original CHARLS was approved by the Ethics Review Board of Peking University (IRB00001052-11,015), and all participants signed an informed consent form at the time of participation.

### 2.2 Data collection

#### 2.2.1 Body mass index

Weight and height were collected at baseline and during follow-up interviews. BMI was calculated as weight (kg) divided by height squared (m^2^). Referring to the Chinese adult standard, underweight was defined as a BMI of less than 18.5 kg/m^2^, normal weight was defined as a BMI between 18.5 and 23.9 kg/m^2^, overweight was defined as a BMI between 24 and 27.9 kg/m^2^, and obesity was defined as a BMI of 28 kg/m^2^ or higher.

#### 2.2.2 Depression assessment

The CES-D10 scores were obtained directly from the CHARLS 2015 contains 10 items, each of which is scored as 0 (rarely or not at all), 1 (sometimes), 2 (most of the time), or 3 (all the time). The total score ranges from 0 to 30, with lower scores indicating lower levels of depressive symptoms. Studies have shown that the threshold of 10 has reasonable sensitivity and specificity for Chinese older adults (21). Therefore, we defined the CES-D10 score as 10 ≥ depression (22).

#### 2.2.3 Socio-demographic factors

Socio-demographic factors included age, gender, education level, marital status, and place of residence. Gender was defined as male or female. Educational level was categorized as ‘illiterate’, ‘primary schools’, ‘middle/high school’, and ‘college and above’. Marital status was defined as married if the participant was currently married and living with a spouse or married but currently separated, and unmarried if the participant was divorced, widowed, or never married to a spouse. Residence was defined as urban or rural.

#### 2.2.4 Behavioral factors

Behavioral factors included social activities, smoking history, alcohol consumption history, sleep quality status, and nighttime sleep duration. Drinking history, smoking history, and social interaction history were categorized as ‘yes’ or ‘no’. Sleep quality was assessed based on responses to ‘my sleep was restless’. Total nighttime sleep duration data were obtained from the question ‘In the past month, how much sleep you got at night (average number of hours in a night)’.

#### 2.2.5 Health status

Based on previous studies and clinical expertise (23–29), the factors selected as possible predictors of depression were history of chronic disease [Hypertension (HTN), Cancer (CA), Chronic Lung Disease (CLD), Heart Disease (HD), Stroke (STK), Arthritis/Rheumatism (AR), Liver Disease (LiverD), Chronic Kidney Disease (CKD), Digestive Disorders (DigestD), or asthma], waist circumference, grip strength, satisfaction with life, self-perceived health, ADL score, vision, Hearing, Pain.

#### 2.2.6 Physical fitness

Physical fitness includes respiratory function (Average of three measurements of peak expiratory flow), grip strength, balance, walking speed, and standing from the physical examination questionnaire.

#### 2.2.7 Blood tests

The blood test indicators were selected from those that have been shown to be more important and relevant in the prediction of depression (30–34), hemoglobin (bl_hgb, g/L), C-reactive protein (bl_crp, mg/L), uric acid (bl_ua, μmol/L), creatinine (bl_crea, μmol/L), blood urea nitrogen (bl_bun, mmol/L), cystatin C (bl_cysc, mg/L), total cholesterol (bl_cho, mmol/L), triglycerides (bl_tg, mmol/L), and low-density lipoprotein cholesterol (bl_ldl, mmol/L).

#### 2.2.8 Variable definition criteria

Chronic diseases and pain were based on self-reported diagnoses and were defined as ‘yes’ or ‘no. Life satisfaction, self-perceived health, vision, and hearing were categorized as ‘good’, ‘fair’, and ‘poor’. Activities of daily living were measured using the Index of Independence in Activities of Daily Living (35) (KatzADL), the CHARLS questionnaire consists of 6 items: eating, dressing, transferring, going to the toilet, bathing, and grooming; ‘No, I do not have any difficulties’ and ‘I have difficulties but I can still do it!’ scored 1, and ‘Yes, I have difficulties and need help’ and ‘I cannot do it’ scored 0; therefore, the total KatzADL score indicates the degree of dependence, with lower scores indicating a higher degree of dependence.

### 2.3 Statistical methods

SPSS 26.0 software was used to process the data, and normally distributed measures were (x¯ ± s)The data were processed using SPSS 26.0 software, with normally distributed measures expressed as (x¯ ± s), non-normally distributed measures expressed as M(P25, P75), and qualitative data expressed as frequency counts and percentages (%). LASSO regression was used to screen for the most valuable characteristic variables first, and then multifactorial logistic regression analysis was used to screen for the relevant influencing factors and to construct the prediction model of the nomograms, and to plot the subjects’ work characteristics (ROC) curves, the calibration curves, and the clinical The predictive ability of the nomogram model was assessed by drawing the ROC curve, calibration curve and clinical decision curve. The maximum missing values of all variables extracted did not exceed 20%, and multiple interpolation was used to deal with missing data (36).

## 3 Results

### 3.1 Participant characteristics

A total of 1,392 obese patients were included in this study. The demographic and clinical characteristics of the participants are listed in Table 1. There were 303 (21.8%) male patients and 1,089 (78.2%) female patients. The prevalence of depression in the obese population was 32.68% (455/1392). Several factors including gender, marriage, pain, grip strength, respiration, bl_crea, health status, chronic liver disease, chronic kidney disease, arthritis and rheumatism, heart disease, digestive disorders, ADLs, hearing, vision, and hours of sleep differed significantly between depressed and non-depressed patients (*p* < 0.01).

**Table 1:** Baseline characteristics of the study population.

Variable	Overall	Non-depression	Depression	*p*
n	1,392	937	455	
Age [mean (SD)]	54.5(12.2)	54.4(11.5)	54.6(13.5)	0.785
Gender (%)				
Female	1,089(78.2)	687(73.3)	402(88.4)	<0.001
Male	303(21.8)	250(26.7)	53(11.6)	
Marital (%)				
Married	1,270(91.2)	877(93.6)	393(86.4)	<0.001
Unmarried	122(8.8)	60(6.4)	62(13.6)	
Education (%)				
Illiterate	86(6.2)	56(6.0)	30(6.6)	0.07
Primary	133(9.6)	84(9.0)	49(10.8)	
Second/high school	76(5.5)	61(6.5)	15(3.3)	
College/Uni+	1,097(78.8)	736(78.5)	361(79.3)	
Residence (%)				
Rural	1,107(79.5)	729(77.8)	378(83.1)	0.027
URBAN	285(20.5)	208(22.2)	77(16.9)	
Pain (%)				
No	950(68.2)	754(80.5)	196(43.1)	<0.001
Yes	442(31.8)	183(19.5)	259(56.9)	
Balance (%)				
Fail	285(20.5)	157(16.8)	128(28.1)	<0.001
Pass	1,107(79.5)	780(83.2)	327(71.9)	
Stands (%)				
Fail	21(1.5)	8(0.9)	13(2.9)	0.008
Pass	1,371(98.5)	929(99.1)	442(97.1)	
grip_strength [mean (SD)]	27.8(9.8)	29.1(10.0)	25.0(8.9)	<0.001
bl_crp [mean (SD)]	3.5(5.8)	3.5(6.3)	3.6(4.5)	0.71
bl_ua [mean (SD)]	5.1(1.4)	5.2(1.4)	4.9(1.3)	0.003
bl_crea [mean (SD)]	0.7(0.2)	0.8(0.2)	0.7(0.2)	0.001
bl_bun [mean (SD)]	14.7(4.0)	14.7(3.9)	14.7(4.0)	0.944
bl_cysc [mean (SD)]	0.8(0.2)	0.8(0.2)	0.8(0.2)	0.027
bl_cho [mean (SD)]	187.8(35.6)	186.5(36.0)	190.4(34.8)	0.056
bl_tg [mean (SD)]	181.2(100.2)	181.3(100.5)	180.9(99.6)	0.933
bl_ldl [mean (SD)]	104.2(29.3)	103.5(29.3)	105.7(29.3)	0.189
bl_hgb [mean (SD)]	13.6(1.9)	13.7(1.9)	13.5(1.9)	0.056
breath [mean (SD)]	293.5(110.8)	308.2(113.3)	263.1(98.9)	<0.001
Hypertension (%)				
No	872(62.6)	593(63.3)	279(61.3)	0.514
Yes	520(37.4)	344(36.7)	176(38.7)	
Diabetes (%)				
No	1,366(98.1)	923(98.5)	443(97.4)	0.205
Yes	26(1.9)	14(1.5)	12(2.6)	
Health (%)				
Fair	685(49.2)	491(52.4)	194(42.6)	<0.001
Good	345(24.8)	290(30.9)	55(12.1)	
Poor	362(26.0)	156(16.6)	206(45.3)	
HTN (%)				
No	850(61.1)	588(62.8)	262(57.6)	0.072
Yes	542(38.9)	349(37.2)	193(42.4)	
CA (%)				
No	1,362(97.8)	923(98.5)	439(96.5)	0.025
Yes	30(2.2)	14(1.5)	16(3.5)	
CLD (%)				
No	1,259(90.4)	867(92.5)	392(86.2)	<0.001
Yes	133(9.6)	70(7.5)	63(13.8)	
HD (%)				
No	1,125(80.8)	780(83.2)	345(75.8)	0.001
Yes	267(19.2)	157(16.8)	110(24.2)	
STK (%)				
No	1,357(97.5)	920(98.2)	437(96.0)	0.027
Yes	35(2.5)	17(1.8)	18(4.0)	
AR (%)				
No	912(65.5)	670(71.5)	242(53.2)	<0.001
Yes	480(34.5)	267(28.5)	213(46.8)	
DL (%)				
No	1,077(77.4)	738(78.8)	339(74.5)	0.087
Yes	315(22.6)	199(21.2)	116(25.5)	
LiverD (%)				
No	1,315(94.5)	882(94.1)	433(95.2)	0.505
Yes	77(5.5)	55(5.9)	22(4.8)	
CKD (%)				
No	1,309(94.0)	902(96.3)	407(89.5)	<0.001
Yes	83(6.0)	35(3.7)	48(10.5)	
DigestD (%)				
No	1,107(79.5)	777(82.9)	330(72.5)	<0.001
Yes	285(20.5)	160(17.1)	125(27.5)	
Asthma (%)				
No	1,337(96.0)	907(96.8)	430(94.5)	0.056
Yes	55(4.0)	30(3.2)	25(5.5)	
ALQ (%)				
No	1,060(76.1)	692(73.9)	368(80.9)	0.005
Yes	332(23.9)	245(26.1)	87(19.1)	
Smoking (%)				
No	1,239(89.0)	817(87.2)	422(92.7)	0.003
Yes	153(11.0)	120(12.8)	33(7.3)	
Sleep quality (%)				
Rarely or none of the time	722(51.9)	618(66.0)	104(22.9)	<0.001
Some or a little of the time	200(14.4)	139(14.8)	61(13.4)	
Occasionally or a moderate amount of the time	206(14.8)	85(9.1)	121(26.6)	
Most or all of the time	264(19.0)	95(10.1)	169(37.1)	
Life_satisfy (%)				
Fair	722(51.9)	424(45.3)	298(65.5)	<0.001
Good	638(45.8)	506(54.0)	132(29.0)	
Poor	32(2.3)	7(0.7)	25(5.5)	
ADL_score [mean (SD)]	5.9(0.4)	5.9(0.3)	5.9(0.5)	<0.001
Waist [mean (SD)]	99.1(11.5)	99.4(10.9)	98.4(12.6)	0.112
Hearing (%)				
Fair	720(51.7)	453(48.3)	267(58.7)	<0.001
Good	544(39.1)	430(45.9)	114(25.1)	
Poor	128(9.2)	54(5.8)	74(16.3)	
Vision (%)				
Fair	517(37.1)	312(33.3)	205(45.1)	<0.001
Good	641(46.0)	490(52.3)	151(33.2)	
Poor	234(16.8)	135(14.4)	99(21.8)	
Sleep hour [mean (SD)]	6.5(1.9)	6.8(1.8)	5.9(1.9)	<0.001

### 3.2 Baseline comparisons results for the training and validation sets

The training (*n* = 974) and validation (*n* = 418) sets showed balanced baseline characteristics for most variables, including age, gender, education, chronic diseases (e.g., hypertension, diabetes), and key predictors like grip strength and respiratory function (*p* > 0.05). Minor differences were observed in alcohol (validation: 27.8% vs. training: 22.2%, *p* = 0.03) and hearing/vision slightly higher in validation, *p* < 0.05, likely due to random sampling variability. Sensitivity analyses confirmed these differences did not affect model performance. Overall, the cohorts are sufficiently comparable, and the model’s validity remains robust. Baseline comparisons and results for the training and validation sets are shown in Table 2.

**Table 2:** Multifactor logistic regression model.

Variable	Multivariate analysis OR(95%CI)	*p*
Age	0.975(0.963–0.987)	<0.001
Grip_strength	0.971(0.953–0.99)	0.003
Breath	0.998(0.996–0.99)	0.013
AR		
Yes	1.676(1.22–2.31)	0.001
No	Reference	
CKD		
Yes	2.077(1.12–3.84)	0.019
No	Reference	
DigestD		
Yes	1.349(0.93–1.94)	0.109
No	Reference	
ADL_score	0.647(0.41–0.99)	0.053
Sleep hour	0.8(0.74–0.87)	<0.001

### 3.3 Depression risk in obese patients: LASSO regression results

**Figure 1:**
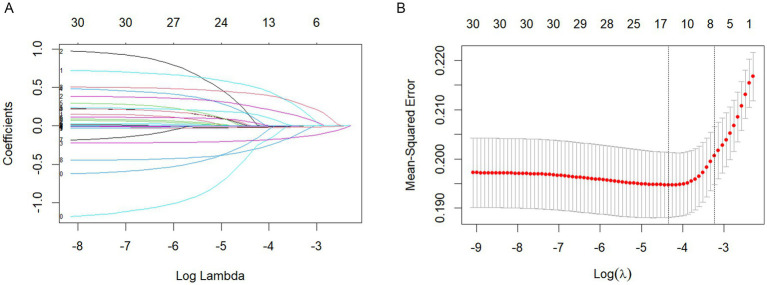
**(A)** LASSO regression model. **(B)** LASSO regression model.

In the LASSO regression model, variables were selected using the 1seλ criterion of cross-validation with MSE. The LASSO regression model was used in Figures 1A,B for demographic and clinical feature selection. Based on the logarithmic (lambda) sequence, a coefficient distribution is generated, and the optimal lambda generates non-zero coefficients. The best parameters (lambdas) in the LASSO model are selected by tenfold cross-validation using the minimum criteria. A partial likelihood bias (binomial bias) curve is plotted relative to the logarithmic (lambda). Draw a virtual vertical line at the optimal value using one SE (1-SE standard) of the minimum standard.

### 3.4 Logistic regression results of depression risk in obese patients

Then, we further used a multivariate logistic regression model for final variable screening. Finally, seven key predictors were identified: age, respiratory function, kidney disease, grip strength, rheumatism and arthritis, ADL score, and sleep duration. See Table 2.

### 3.5 Results of the depression risk prediction model for obese patients

**Figure 2:**
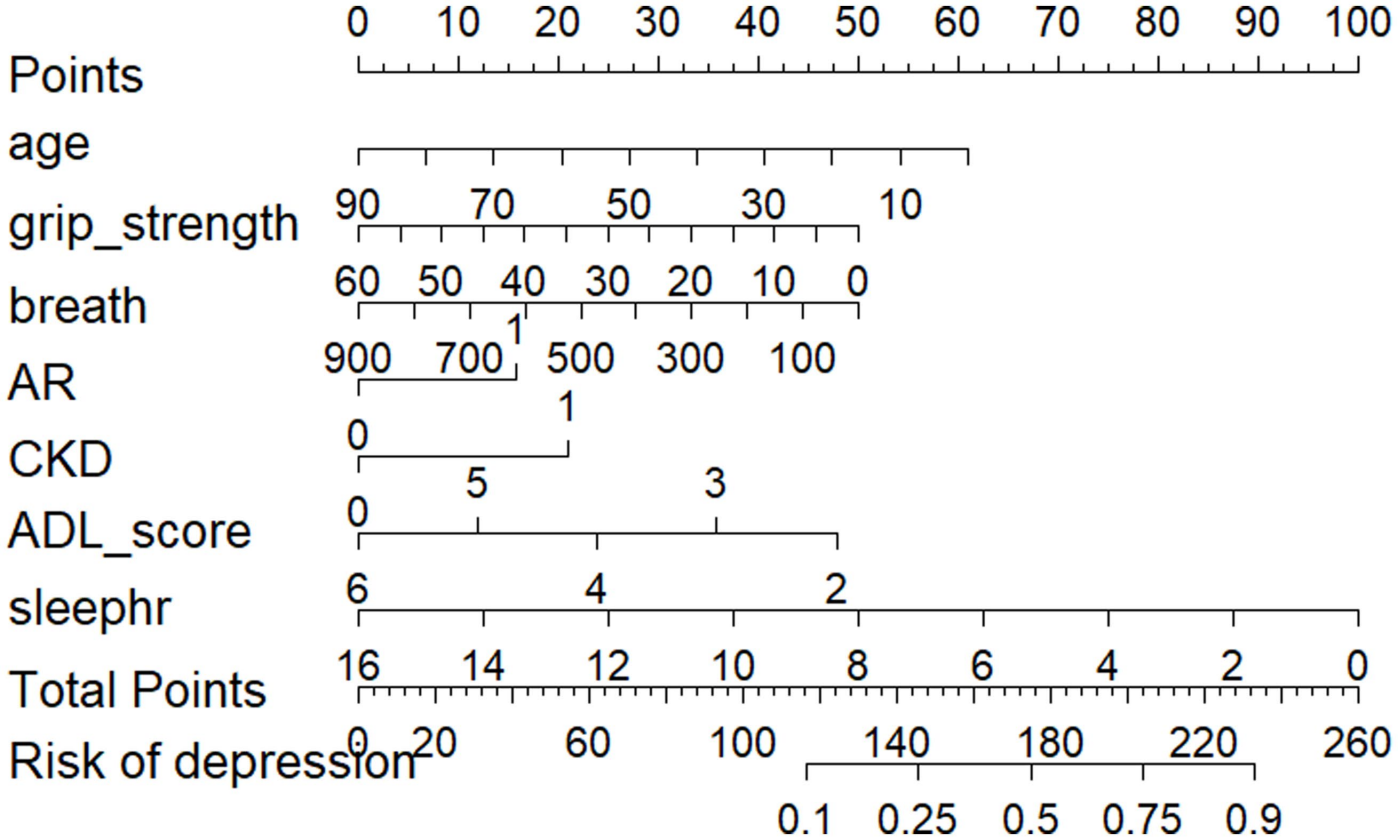
Nomogram of the prediction model.

The predictive model consists of variables with a *p* value of less than 0.05 in multivariate logistic regression. These variables include age, respiratory function, kidney disease, grip strength, rheumatism and arthritis, ADL, and sleep duration as predictors. The predictive model is represented using a nomogram. For each feature in the nomogram, find the corresponding value. Draw a vertical line from that value to the top ‘Points’ scale to determine the score for that characteristic. Calculate Total Score: Add up all the scores for each characteristic to get a ‘total’ score. Estimate Risk: Match the total score with the ‘Risk’ scale at the bottom of the chart to get an estimated probability of risk. For example, a 50-year-old patient with respiratory dysfunction (PEF = 300 L/min), a grip strength of 60 kg, arthritis and renal disease, an ADL score of 4, and 8 h of sleep for a total score of 180 equates to a 50% risk of depression which can be used to quantitatively predict the risk of depression in obese patients (Figure 2).

### 3.6 Validation of depression risk prediction model for obese patients

**Figure 3:**
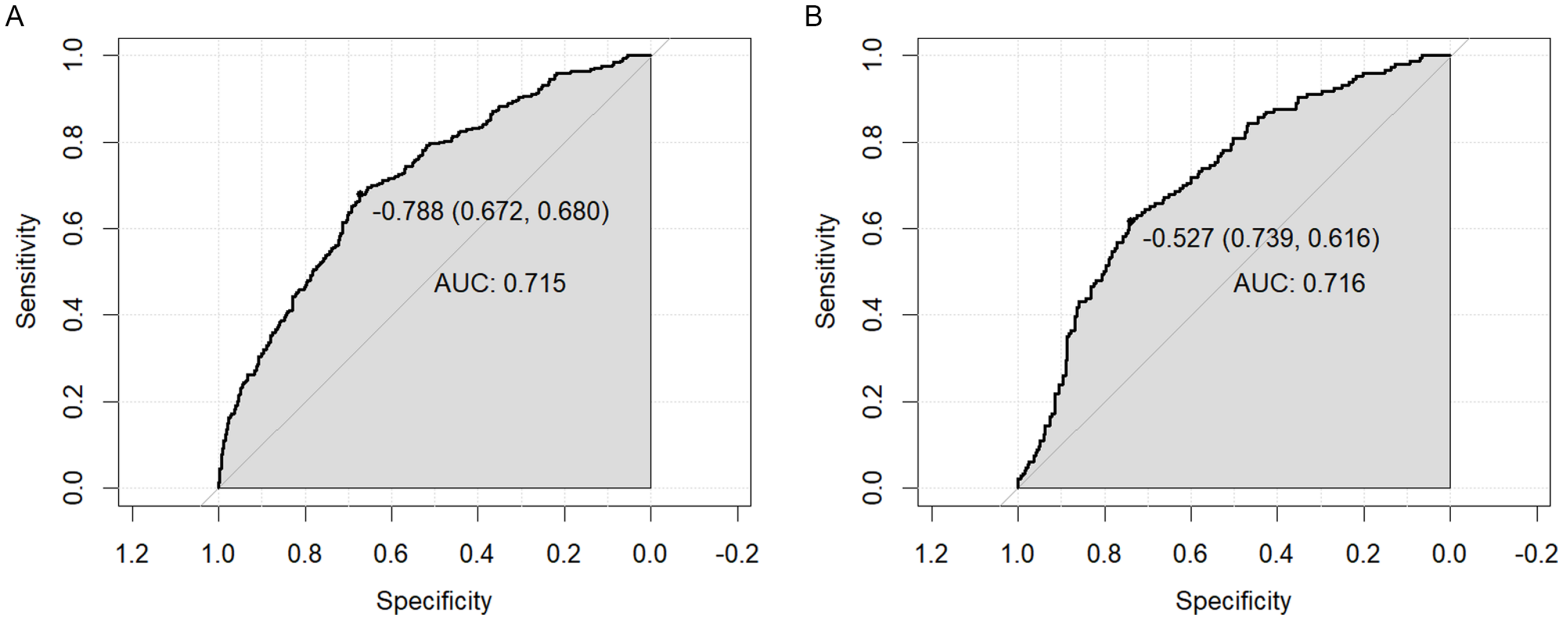
**(A)** AUC curve of the training set. **(B)** AUC curve of the test set.

AUC values were calculated to assess the discriminatory performance of the predictive model by examining the occurrence of depression in obese patients in the training set and validation set. As shown in Figures 3A,B, the area under the ROC curve of the predicted model in the training set was 0.715 (95% CI = 0.681–0.749), specificity was 0.672, sensitivity was 0.680, AUC = 0.716 (95% CI = 0.665–0.767), specificity was 0.739, and sensitivity was 0.616, in the validation set. These data suggest that nomograms have good discriminative ability and predictive value and can correctly identify depressed and non-depressed patients.

### 3.7 Calibration of depression risk prediction model for obese patients

**Figure 4:**
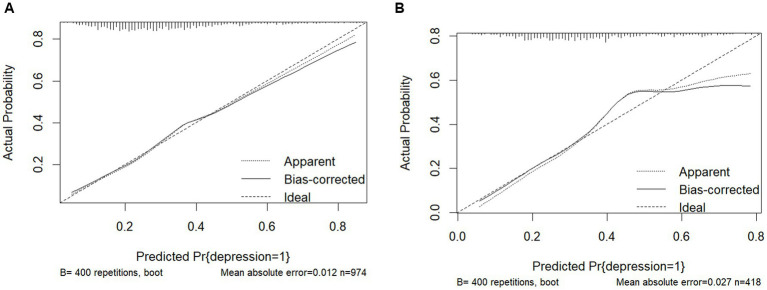
**(A)** Training set calibration curve. **(B)** Test set calibration curve.

The nomogram were evaluated using calibration plots and the Hosmer-Leme show goodness-of-fit test (*p* > 0.05 indicating that the model exhibited a very good fit). The test results show that the calibration curve is a straight line close to 1 and that the model fits very well for both the training (χ^2^ = 5.44, df = 8, *p* = 0.931) and validation sets (χ^2^ = 13.45, df = 8, *p* = 0.926). The calibration plots of the training and validation sets based on the multifactor Logistic regression model are shown in Figures 4A,B. The calibration curves of the columnar plots show a high degree of agreement between the predicted probability of depression in training and the actual probability of Figure 4A, and validation sets (Figure 4B).

### 3.8 Evaluation of the clinical validity of the depression risk prediction model in obese patients

**Figure 5:**
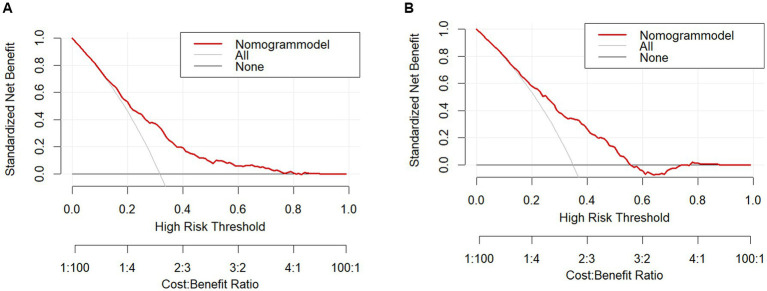
**(A)** Training set decision curve. **(B)** Test set decision curve.

The clinical validity of the models was assessed using the DCA method, and the results are shown in Figures 5A,B. From the decision curves, the net gain of the predictive model for the internal validation set was significantly higher than the two extreme cases, indicating that the nomogram graph model has better net gain and predictive accuracy.

## 4 Debate

In this study, the prevalence of depression in the obese population was 32.68% (455/1392), which was significantly higher than the prevalence among the general population in China (37), which may be due to the existence of common biology between the two (8), thus increasing the prevalence of depression in the obese population. In the present study, variables were screened by LASSO regression and multifactorial logistic regression, and the results showed that age, respiratory function, ability to perform activities of daily living, grip strength, arthritis and rheumatism, renal disease, and sleep duration were independent influences on depression in the obese population. While the nomogram includes variables accessible in clinical settings (e.g., grip strength, sleep duration), some indicators (e.g., renal function) require medical evaluation. Therefore, this tool is primarily intended for clinicians to screen high-risk obese patients, rather than self-assessment by individuals.

Impairment of respiratory function may lead to cerebral hypoxia and metabolic disorders (38), which in turn triggers an inflammatory state in which the body releases a large number of pro-inflammatory factors and the level of systemic inflammatory response increases, leading to the development of depressive symptoms (39). Relevant studies have shown that a good state of respiratory function is conducive to the prevention of depression (40), at the same time, the risk of depression in people with abnormal respiratory function is significantly higher (41, 42), in addition, respiratory training for respiratory function can effectively improve the respiratory function and depressive symptoms, reducing the risk of depression (43–45). Therefore, obese patients should pay attention to respiratory function, and patients with decreased respiratory capacity should undergo respiratory training as early as possible to reduce the risk of depression and help prevent depression.

The results of this study showed that lower grip strength was associated with depression, which is consistent with the results of several related studies (46). Grip strength reflects muscle strength to some extent. When a muscle contracts, the body releases a variety of bioactive substances, including irisin. Irisin is a peptide hormone secreted by muscles, which can act on the brain to regulate the neuroendocrine system and promote neurogenesis in the hippocampus, a brain region closely related to emotion regulation and memory. The hippocampus of depressed patients often suffers from atrophy and dysfunction, and appropriate grip strength training can increase irisin secretion, which helps to improve the function of the hippocampus, which may be one of the potential mechanisms for its improvement of the depressive state (47). Meanwhile, changes in grip strength may also affect the activity of the hypothalamic–pituitary–adrenal (HPA) axis (48). Prolonged stress or depression can lead to dysfunction of the HPA axis, resulting in abnormal secretion of stress hormones such as cortisol. Exercise training can regulate the body’s stress response, make the secretion of cortisol more rational, and alleviate depression-related neuroendocrine abnormalities (49), and grip strength, as a manifestation of muscle strength, may have a similar regulatory effect. Meanwhile, since HGS is a simple, non-invasive and inexpensive measure, it can be easily used in clinical practice to test patients help to identify those at higher risk of mental health problems early (50), and grip strength training for obese people with low grip strength to effectively reduce the risk of developing depression.

The results of this study showed that age was associated with the incidence of depression, i.e., age was negatively correlated with depression in the obese population, which may be due to the fact that adolescents are in the stage of rapid physical and mental development and are highly concerned about their self-image compared with the middle-aged and older adult population. In the face of pressure brought by obesity, such as social discrimination and health problems, they are prone to depression due to external evaluations, and several systematic evaluations have confirmed that obesity significantly increases the risk of depression in adolescents (51, 52). In contrast, middle-aged and older adults may be more likely to view others’ perceptions of their obese body size more favorably, thus reducing the likelihood of obesity-induced dysphoria. Studies have confirmed that among middle-aged and older adults, obese patients are relatively more likely to suffer from depressive symptoms (21, 53). This is consistent with our findings.

Our predictive model showed that low ADL scores were also associated with depression. Several studies have shown that limited physical functioning leads to an increased prevalence of depression (54–56). Individuals with low ADL scores tend to be less socially active as a result of limited physical activity, have less contact and communication with the outside world, and tend to fall into loneliness and self-isolation, which is one of the important risk factors for depression. A previous cross-sectional study also demonstrated the relationship between loss of functioning and depression and further confirmed that the ADL score is a predictor of depression (55). ADL reflects an individual’s ability to take care of him/herself. Individuals with impaired ADL usually feel helpless and frustrated and have low self-esteem, and being in this state for a long period tends to trigger depressive moods. In addition, a large number of studies have included ADL scores in risk prediction models for depression (57–59), which reinforces that ADL scores are an important predictor of depression risk.

Arthritis and rheumatic diseases are a large group of diseases that involve the joints and their surrounding tissues, and there is a complex association between them and depression. They cause chronic pain that constantly stimulates nerves, which are transmitted via peripheral nerves to the central nervous system. The constant pain signaling affects the balance of neurotransmitters and leads to depression. In addition, chronic pain in patients with arthritis and rheumatism often causes sleep problems. Sleep deprivation or sleep disruption interferes with the neuroendocrine system, affecting the normal secretion of hormones, such as cortisol, and causing the production of adverse moods. A large cohort study from Canada (60) showed that 1 in 10 people with arthritis had a level of major depression and that people with arthritis had higher levels of depression compared with people without arthritis. Therefore, it is important to pay particular attention to the mood of patients with arthritis and rheumatism in the obese population to reduce the risk of depression.

Kidney disease is a common chronic disease; depression is especially common in chronic kidney disease (61–63). In different stages of kidney disease development, there are different degrees of depression and anxiety changes (64). The development of the disease will increase the related complications, which will seriously affect the quality of life of the patients and bring a great impact on the mental health of the patients, thus developing into depression.

Sleep duration: At present, studies in several countries have shown that a short sleep duration is significantly associated with an increased risk of developing depression (65, 66). Sleep is crucial for the regulation of neurotransmitters, and the lack of sleep causes abnormal expression of neurotransmitters such as GABA, NPY, and 5-HT, and when inhibitory neurotransmitters are at low levels, it causes the patient’s brain to be in a depressed state, reducing the patient’s expression of emotion and the emergence of a depressive state. In addition, sleep has an important regulatory role in the function of the hypothalamic–pituitary–adrenal (HPA) axis (67). Normal sleep helps to maintain the normal rhythm of the HPA axis and keeps the secretion of stress hormones, such as cortisol, at a reasonable level. Reduced sleep duration leads to dysfunction of the HPA axis and an abnormal increase in cortisol secretion. Prolonged exposure to high cortisol can have adverse effects on the brain, such as damaging brain areas related to memory and mood regulation, such as the hippocampus, thereby increasing the risk of depression. Therefore, when dealing with obese patients with sleep problems, it is necessary to keep an eye on their mental health and be alert to negative emotions to prevent the development of depression.

Nomograms are commonly used as predictive models in many clinical areas of research. Predictive models based on nomograms have the characteristics of high accuracy, simplicity, and practicality. In this study, LASSO regression was used in combination with multifactor logistic regression analysis to screen seven variables that were highly correlated with depression, which reduced the problem of multicollinearity among factors and further improved the accuracy of model prediction. Although the AUC values (0.715–0.716) indicate moderate discrimination, the model achieves a balanced sensitivity (68.0%) and specificity (73.9%). The calibration curves show that there is significant agreement between the nomogram model and the actual observations. Furthermore, the DCA curve and net gain curve of the model indicate that the model has a high gain in predicting depression, supporting its utility in clinical screening.

There are also some limitations of this study. First, although the CES-D-10 is a commonly used tool for assessing clinically meaningful depressive symptoms, the CES-D-10 was used to examine the number of self-reported depressive symptoms in the past week, which may introduce recall bias. Second, the nomogram was developed based on data from China, and whether the results of this study can be extended to other regions and countries needs to be further validated using data from external cohorts. This study did not account for genetic predisposition or acute psychosocial stressors, which may influence depression risk. Future models should integrate these factors to validate the model in a multicenter cohort to improve predictive accuracy.

## 5 Conclusion

This study developed and validated a nomogram model that can predict the risk of depression in obese patients, conclusively identifying age, respiratory function, renal disease, digestive disease, grip strength, rheumatism and arthritis, and sleep duration as risk factors for the development of depression in the obese population. This may provide assistance to clinicians in screening at-risk populations and optimize personalized prevention strategies for healthcare professionals.

## Data Availability

The datasets presented in this study can be found in online repositories. The names of the repository/repositories and accession number(s) can be found at: http://charls.pku.edu.cn.
