# Correction: Development and validation of a predictive model for depression risk in Chinese obese adults

**DOI:** 10.3389/fpubh.2025.1735400

**Published:** 2025-11-17

**Authors:** Cong Yu, Jiamin Cao, Wenguang Chen, Ensi Hong

**Affiliations:** 1 Clinical Medical College, Jiangxi University of Chinese Medicine, Nanchang, China; 2 Nanchang Normal University, Infirmary of the Logistics Support Department, Nanchang, Jiangxi, China; 3 The Affiliated Hospital of Jiangxi University of Chinese Medicine, Nanchang, China

**Keywords:** obesity, risk of depression, predictive models, nomogram, Chinese population

Affiliation 1 “Clinical Medical College, Jiangxi University of Chinese Medicine, Nanchang, China” was erroneously given as “Graduate School, Jiangxi University of Chinese Medicine, Nanchang, China”.

Author Ensi Hong is only affiliated to affiliation 3 “The Affiliated Hospital of Jiangxi University of Chinese Medicine, Nanchang, China”. Affiliation 1 was removed from Ensi Hong.

The original version of this article has been updated.

